# Attachment as a mechanism influencing end-of-life communication: An analogue investigation

**DOI:** 10.1371/journal.pone.0303652

**Published:** 2024-07-31

**Authors:** Holly E. Evans, Ursula M. Sansom-Daly, Richard A. Bryant

**Affiliations:** 1 School of Psychology, UNSW Sydney, Sydney, New South Wales, Australia; 2 School of Clinical Medicine, UNSW Medicine & Health, Randwick Clinical Campus, Discipline of Paediatrics, University of New South Wales (UNSW) Sydney, Randwick, New South Wales, Australia; 3 Behavioural Sciences Unit, Kids Cancer Centre, Sydney Children’s Hospital, Sydney, New South Wales, Australia; 4 Sydney Youth Cancer Service, Nelune Comprehensive Cancer Centre, Prince of Wales Hospital, Randwick, New South Wales, Australia; Fondazione Policlinico Universitario Agostino Gemelli IRCCS, Universita’ Cattolica del Sacro Cuore, ITALY

## Abstract

Talking about dying when faced with end-of-life may be important for achieving optimal outcomes for young people and their families. Given the lack of research on young people’s communication around end-of-life and death, this analogue study examined the role of attachment theory in conversations about dying. Experiment 1 assessed attachment security of 80 healthy young adults and randomised them to receive either an induction that raised awareness of one’s attachment figures or a neutral induction, and then primed them with an imagined scenario where they were diagnosed with an incurable illness. Participants then completed a self-report measure of their willingness to discuss end-of-life topics with family, friends, or a psychologist. The experimental attachment induction did not increase willingness to talk about end-of-life concepts. Experiment 2 extended this design and asked participants to describe these conversations and assessed the content of their imagined end-of-life conversations. Experiment 2 replicated the finding that enhancing individuals’ awareness of key attachment figures did not increase participants’ willingness to engage in end-of-life conversations. However, heightened attachment awareness led participants to talk more about their relationship with the person they were hypothetically talking with. Across both experiments, avoidant attachment tendencies reduced the likelihood that participants receiving the attachment prime would want to engage in end-of-life conversation. Overall, it seems there are important differences between individuals on willingness to talk about death, and this may be influenced by one’s attachment style. These results raise implications for the importance of attachment in the therapeutic relationship for healthcare professionals working with young people with life-limiting illnesses, such as cancer. Further research may shed light on how an individually tailored approach, taking into account attachment security, achieves the best outcomes for individuals who require end-of-life conversations.

## Introduction

Cancer is a leading cause of death from disease for adolescents and young adults [AYAs) in many countries [1]. For these young people with cancer and other life-limiting illnesses, there is usually a period where it is known that the young person is unlikely to survive. This means there is often time for their treating healthcare team, family, and the young person to prepare for death and consider end-of-life care. Good psychosocial care for AYA cancer patients at end-of-life is important not only for the patient’s psychological adaptation to death, but also for the ongoing mental health of the bereaved family [2, 3]. Recent guidelines that have outlined standards for psychosocial palliative care for dying AYAs have identified effective, age-appropriate communication at end-of-life as important [4, 5].

Despite the considerable attention given to end-of-life conversations, these have predominantly focused on strategies to optimize communications between health professionals and patients. Numerous reviews have identified a range of communication factors that potentially contribute to these conversations, including rapport, conversational skills, family involvement, tailoring of information, empathy, and revisiting goals of care [6, 7]. Interestingly, meta-analyses indicate that the various strategies studied to optimize end-of-life conversations do not significantly impact occurrence of these communications [8]. This highlights the need for more attention on fundamental mechanisms underpinning end-of-life conversations, and particularly the role of relationships with health professionals and family.

Research on end-of-life communication with AYAs with cancer has mainly focussed on factors influencing communication from the perspective of healthcare professionals. Evidence suggests that healthcare professionals often report feeling ill-equipped to communicate with AYAs about their prognosis, preferences for care and final wishes in developmentally appropriate ways [9, 10]. While this information may be helpful to improve the competency of healthcare professionals in these conversations, there is a dearth of research on end-of-life communication from the young person’s perspective. The young person’s perspective is particularly important, as communication is a two-party process, and being guided by the perspectives of patients is critical for patient-centered healthcare [11]. This is also consistent with best-practice models of care for AYA cancer patients, which emphasise the importance of supporting the young person’s autonomy and active involvement throughout their interactions with the health system [12]. Evidence suggests that AYAs generally do want to be involved in end-of-life conversations, and that they have preferences for how these conversations happen [13]. However, other research suggests that AYAs vary in their level of engagement with end-of-life conversations, with some studies of both healthy and chronically-ill AYAs finding that some young people do not wish to be involved in end-of-life decision-making at all [14, 15].

There is a large gap in the literature on psychological factors influencing individual differences in when and how AYAs talk about their own death. Given this lack of research, it may be useful to consider other theories related to coping with threat. One candidate theory that has potential to address communication about end-of-life is attachment theory. According to this theory, the attachment system is established in infancy, driving children to seek proximity to caregivers (i.e., attachment figures) when faced with emotional or physical threat [16]. The responses children receive from their caregivers throughout critical years of their development shapes what they ‘learn’ about the availability of attachment figures later in life. In adults, attachment security has been conceptualised as an emotional resource to be used in physically or emotionally threatening situations, bolstering coping ability by encouraging an individual to seek comfort from supportive people [17]. Supporting this proposal is much evidence that both proximity and awareness of one’s attachment figures can reduce stress responses on behavioural, cognitive, and neural indicators [18–20], including via imaginal inductions [20], and the physical presence of one’s attachment figure [19, 21].

However, security of adult attachment varies across individuals, with insecurely attached people typically conceptualized as either anxious or avoidantly attached. Anxiously attached people believe that social support is unavailable or inconsistent, and in response intensify proximity-seeking behaviours to seek out their attachment figure’s attention (*hyperactivation* of the attachment system). Avoidantly attached individuals also tend to believe that social support is inconsistent or unavailable, but respond by maintaining independence and emotional distance, coping on their own in times of stress (*hypoactivation* of the attachment system) [17]. Numerous studies have found that one consequence of being insecurely attached is that it results in a diminished ability to benefit from attachment figures during threat [22–24], which is supported by evidence that insecurely attached individuals do not benefit as much from attachment security inductions as people with secure attachment systems [22–24].

The current experiments aimed to investigate how attachment tendencies may influence end-of-life communication outcomes among a sample of healthy young adults; this population was chosen due to ethical considerations inherent with testing experimental theories on a vulnerable and burdened population such as young cancer patients and survivors. Attachment security may enhance end-of life communications because it may (a) reduce stress associated with discussing a potentially distressing topic, (b) promote trust in others that can facilitate discussions, and (c) boost self-esteem that can promote a sense of confidence in engaging with other people in discussing end-of-life. Here we examined the role of attachment by examining both transient changes in attachment system activation, as well as trait individual differences in attachment security. Across two experiments, we manipulated the extent to which individuals were aware of attachment figures by priming them with awareness of personal attachment figures (attachment system activation) and measured how this impacted willingness to engage in analogue end-of-life conversations. We also investigated the influence of individual attachment style on responses to the attachment primes and willingness to engage in these analogue end-of-life conversations.

## Experiment 1

Experiment 1 aimed to test the hypothesis that attachment priming may promote greater willingness to engage in end-of-life conversation with trusted others. On the premise that people gravitate towards close others, we hypothesised that priming awareness of attachment figures would facilitate end-of-life conversation with a trusted/close individual. As reliance on others also depends on individual differences in attachment style, we hypothesised this relationship to be moderated by the level of attachment security such that those with insecure attachment styles may be less likely to be influenced by the attachment prime. Specifically, we hypothesised that being reminded of a close attachment figure through an experimental attachment induction would increase participants’ willingness to talk about end-of-life, relative to a neutral control induction. Further, we hypothesised this effect would be lessened in people with more anxious and avoidant attachment tendencies (i.e., who had less secure attachment styles).

## Materials and methods

### Participants

Participants were 87 first-year psychology students aged 17–25 years participating in return for course credit, which was deemed by the ethics committee to not represent an excessive inducement. The experiment was approved by the University of New South Wales Human Research Ethics Advisory Panel C. Participants registered for the study via an online research portal, where they were told that the study would involve thinking about future life events including illness and death. All participants gave written informed consent. Due to ethical issues regarding imagining one’s end-of-life, participants were only eligible to participate if they scored below the severity cut-off for depression [11] or anxiety [8] on the Depression Anxiety and Stress Scales 21-item (DASS-21] [25]; participants scoring above these cut-offs were excluded following DASS completion and prior to the experiment. Participants were enrolled between July 18, 2018 and September 12, 2018.

### Measures

#### Depression, anxiety, and stress symptoms

We used the DASS-21 [25] to assess depression, anxiety, and stress over the past week. This measure has strong psychometric properties to assess anxiety and depression [26], including in adolescents and young adults [27].

#### Manipulation checks

To assess whether the experimental induction led to an impact on perceived distress, positivity, and closeness affect ratings as well as vividness of visualisation were completed before and after the attachment induction, as well as after an end-of-life visualisation task, to enable us to assess the function of the independent variables. Each scale was rated on 100-point scale (e.g., 1 = *not at all distressed*, 100 = *extremely*). For example, “How close do you feel to others right now on a scale of 1 (not at all close) to 100 (extremely close)?”

#### Attachment style

Participants completed the Experience in Close Relationships Scale—Revised (ECR-R) [28] that comprises two subscales that index anxious and avoidant attachment tendencies, respectively. This is a 36-item self-report scale on which items are rated on a 7-point scale (1 = *strongly disagree*, 7 = *strongly agree*). Higher scores on either subscale indicate more severe anxious and avoidant attachment tendency, respectively. The ECR-R has good internal consistency of 0.94 and 0.93, respectively, for the anxiety and avoidance attachment constructs [29].

#### Willingness to talk

Willingness to talk to friends/family was measured by four questions that enquired into their willingness to engage with these figures, in terms of likeliness of talking and comfort talking to others. Another two questions were similarly structured but referred to discussing end-of-life issues with a psychologist (e.g., ‘How likely would you be to talk to a psychologist (/family member/friend) about end-of-life, on a scale of 10-point scale (1 = *not at all likely*, 10 = *extremely likely*). An average score was calculated for these items, which yielded adequate internal consistency (Cronbach’s Alpha = .71).

### Procedure

Participants initially completed informed consent and the DASS-21. They were then randomly assigned to complete either the attachment or control induction, in which they were instructed to imagine either a supportive person (attachment induction) or a neutral acquaintance (control induction) for three minutes. Participants in the attachment condition were directed to think of *“someone in your life who is very supportive to you*. *The person who you would turn to when you need help*. *Someone who is very close to you and has been there for you when you needed them*.*”* Participants in the control condition were given the same instructions that pertained to a neutral person whom they knew but they were not close to. They were asked to close their eyes and visualise the person and were verbally prompted to continue every 30 seconds (e.g., *“Keep imagining that person*.*”*). This is a proven induction that has been shown to reliably induce affinity to attachment figures [20, 30, 31].

Participants were then read a script by the experimenter, asking them to visualise having incurable cancer, outlining things they may be concerned about such as emotional impacts, missing out on future life events, impact on friends and family, and questions surrounding the afterlife. A portion of the script is given below.

*Imagine being told that you have cancer that can’t be cured, and that you are going to die from the disease. It’s a horrible reality to face, especially when you’re young. You have to deal with so many emotions; anger, sadness, fear, guilt.*

After the script was read, participants were asked to visualise the scenario for a further two minutes. They then answered the set of questions about willingness to engage in conversations about end-of-life.

Manipulation checks were administered before the attachment/control induction, after the induction, and after the end-of-life visualisation.

The first author was aware of participants’ identities, and these were stored in de-identified forms in the primary datasets prior to being available to other members of the research team.

### Analysis

Following a previous study which demonstrated a moderate effect of attachment activation on response to a stressor [24], we estimated a required sample size of at least 35 per cell to achieve an effect size of 0.6 between the two conditions, providing power of 80% to detect a difference between conditions at the 5% significance level.

All analyses were performed using SPSS Version 25. Planned comparisons and repeated measures analyses of variance (ANOVAs) were used to test the differences between attachment and control groups on the manipulation checks. To test the between-group relationships between willingness to talk about end-of-life and attachment security, three moderation analyses were performed using the PROCESS macro v.3 (Model 2). These moderation analyses were conducted with group (attachment vs control) as the predictor variable, ECR-R attachment, and avoidant subscales as the moderator variables, and willingness to talk as the outcome variables. We conducted separate moderation analyses with willingness to talk to friends/family, talking to a psychologist, and talking to friends/family/a psychologist as the outcome variable.

## Results and discussion

### Participant characteristics

Participant characteristics are presented in Table 1. Seven participants were excluded due to scores above the screening cut-offs on the DASS-21 Anxiety or Depression scales. After excluding these participants, there were 41 participants in the attachment condition, and 39 in the control condition. There were no significant differences between induction conditions on age, DASS-21 subscale scores, or ECR scores. However, females were significantly more likely to be in the attachment group than the control group [*χ*^*2*^(1, *N* = 80) = 4.27, *p* = .039]. Group means fell within normal ranges for DASS-21 Depression, Anxiety and Stress subscales.

**Table 1:** Experiment 1 participant characteristics.

	Attachment mean *(SD)*	Control mean (SD)
Age	20.69 *(5*.*37)*	20.58 *(4*.*99)*
Gender (percentages)	78% female	56% female
DASS-21		
Depression	3.93 *(3*.*10)*	2.95 *(3*.*19)*
Anxiety	3.83 *(3*.*35)*	3.90 *(3*.*15)*
Stress	6.19 *(3*.*58)*	5.02 *(3*.*71)*
ECR-R		
Anxious	3.81 *(1*.*05)*	3.62 *(*.*89)*
Avoidant	2.74 *(*.*79)*	3.04 *(*.*94)*
	Attachment N *(%)*	Control N *(%)*
Education level attained		
Post graduate degree	1 *(2*.*4)*	2 *(5*.*1)*
University degree	1 *(2*.*4)*	5 *(12*.*8)*
Technical certificate/diploma, college	4 *(9*.*8)*	1 *(2*.*6)*
Year 12 (the final year of high school in Australia)	35 *(85*.*4)*	31 *(79*.*5)*
Language other than English spoken at home		
No, English only	15 *(36*.*6)*	11 *(28*.*2)*
Yes	26 *(63*.*4)*	28 *(71*.*8)*

*Note*: SD, standard deviation; DASS-21, Depression Anxiety Stress Scale 21-item; ECR-R (higher scores = higher insecure attachment, score has a minimum of zero and maximum of seven), Experiences in Close Relationships Scale–Revised.

### Manipulation checks

There were no differences between the attachment (M = 79.61, SD = 16.78) and control groups (*M* = 77.28, *SD* = 15.05) on vividness of the attachment/control induction (t(78) = -.652, *p* = >.05), indicating that despite the difference in content, the inductions were visualised to similarly vivid and detailed degrees.

A 2 (Induction Condition) x 2 (Time) repeated measures ANOVA on closeness ratings indicated a marginally significant main effect for Time [*F*(1,78) = 4.39, *p* = .053, η_p_^2^ = .053], indicating that participants in both groups perceived marginally greater closeness to their imagined attachment figure over time during the experiment. There was also a significant interaction effect [*F*(1,78) = 5.41, *p* = .023, η_p_^2^ = .065], see Table 2. This interaction indicated that ratings of closeness decreased at greater rate for the attachment group relative to controls. This indicates that the impact of the attachment induction may not be very long-lasting.

**Table 2:** Experiment 1 means and standard deviations for manipulation checks.

	Group	
	Attachment mean *(SD)*	Control mean (*SD*)
Closeness		
Post-induction	70.85 *(19*.*71)*	62.62 *(19*.*08)*
Post-visualisation	59.51 (*23*.*63*)	63.21 (*23*.*34*)
Distress		
Pre-induction	20.59 (*22*.*94*)	22.77 (*26*.*07*)
Post-induction	9.76 (*11*.*81*)	19.00 (*21*.*62*)
Post-visualisation	33.68 (*24*.*52*)	33.28 (*25*.*02*)
Positivity		
Pre-induction	66.71 (*21*.*18*)	64.77 (*20*.*85*)
Post-induction	79.59 (*16*.*32*)	68.49 (*17*.*97*)
Post-visualisation	57.05 (*20*.*39*)	58.33 (*20*.*78*)

A 2 (Induction Condition) x 3 (Time) repeated measures ANOVA on distress ratings indicated a significant main effect for Time [*F*(2,156) = 40.01, *p* = < .001, η_p_^2^ = .339]. Contrasts revealed significant differences between distress pre and post induction [*F*(1,78) = 14.73, *p* = < .001, η_p_^2^ = .159], and between post induction and post end-of-life visualisation [*F*(1,78) = 71.45, *p* = < .001, η_p_^2^ = .478], see Fig 2 and Table 2. This indicates that across groups, distress decreased significantly from baseline to post-induction (after either the attachment/control induction), and then increased significantly from that point until after the end-of-life visualisation.

There were significant differences across the positivity ratings [*F*(2,156) = 33.98, *p* < .001, η_p_^2^ = .303]. Contrasts again revealed significant differences between positivity at pre- and post-induction [*F*(1,78) = 18.66, *p* < .001, η_p_^2^ = .193] and between post-induction and post-visualisation [*F*(1,78) = 67.03, *p <* .001, η_p_^2^ = .462]. There was also a significant interaction between positivity and group [*F*(2,156) = 5.25, *p* = .006, η_p_^2^ = .063]. Further contrasts were performed comparing each timepoint of positivity scores between groups. There were significant interactions at pre-induction compared to post-induction [*F*(1,78) = 5.69, *p* = .020, η_p_^2^ = .068] and at post-induction compared to post-visualisation [*F*(1,78) = 9.62, *p* = .003, η_p_^2^ = .110]. This pattern suggests that individuals who received the attachment prime experienced a greater increase in positivity ratings than their control counterparts, but that when both groups were asked to think about their own death in the end-of-life visualisation this between-group difference was reduced.

### Willingness to engage in end-of-life conversation

For the first moderation analysis using talking to friends/family as the outcome variable, the priming condition did not independently predict willingness to talk [*b* = -.40, *t*(74) =.-.20, *p* = .842]. The mean for the attachment group was 8.29 [*SD* = 1.89], while the mean for the control group was 7.14 [*SD* = 2.29].

For the second moderation analysis using talking to a psychologist as the outcome variable, the priming condition did not independently predict willingness to talk [*b* = 1.57, *t*(74) = .63, *p* = .533]. The mean for the attachment group was 6.63 [*SD* = 2.38], while the mean for the control group was 6.32 [*SD* = 2.46]. However, the interaction with avoidant attachment style was significant [*F*(1, 74) = 6.55, *p* = .020, change *R*^*2*^ = .061], indicating that more avoidantly-attached individuals felt less willing to talk when provided with the attachment prime.

**Fig 1:**
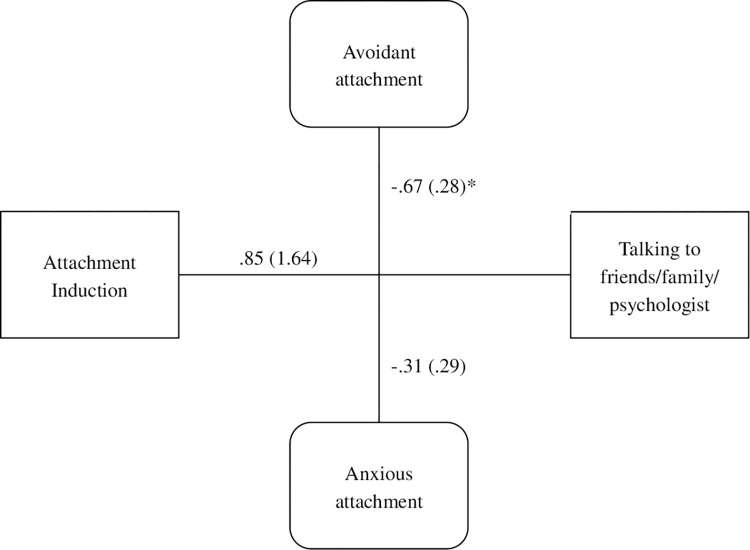
Experiment 1 moderation model of attachment induction, talking about end-of-life with all conversation partners, anxious attachment, and avoidant attachment.

For the final moderation analysis, the priming condition did not independently predict willingness to talk to anyone [*b* = .85, *t*(74) = .52, *p* = .605]. The mean for the attachment group was 7.77 [*SD* = 1.43], while the mean for the control group was 6.91 [*SD* = 1.83]. However, the interaction with avoidant attachment style was significant [*F*(1, 74) = 4.05, *p* = .017, change *R*^*2*^ = .004], indicating that more avoidantly-attached individuals felt less willing to talk when provided with the attachment prime, see Fig 1.

Overall, the attachment induction did not lead participants to express more willingness to have conversations about end-of-life. However, those with higher attachment avoidance were more reluctant to talk about death following the attachment prime. This is consistent with evidence that avoidantly attached individuals tend not to rely on others for social support in difficult situations [24]. Consistent with this notion, numerous studies have found that avoidantly attached people do not respond to attachment priming insofar as they do not display the psychological benefits typically evident when one has attachment figures available.

A further question arises concerning whether priming attachment induction may impact the content of what participants want to talk about. One might expect that if priming representations of attachment figures influences comfort with end-of-life conversations for some, it might also influence the content of those conversations. There is no research to date experimentally manipulating end-of-life conversations and measuring subsequent content of those conversations, particularly in relation to attachment, and accordingly this was the goal of Experiment 2.

## Experiment 2

Experiment 2 aimed to replicate the finding from Experiment 1 that avoidant attachment moderates the impact of attachment priming on motivation to engage in an end-of-life conversation. Further, Experiment 2 aimed to determine the extent to which an attachment induction influences the *content* of end-of-life conversations. To address this issue, we replicated the design of Experiment 1 but included a writing task that asked participants to write as if they were speaking to a close friend or family member in the context of the hypothetical end-of-life scenario. We focused on focus on conversation with a friend or family member on the basis that participants may feel more willing to speak freely with a familiar conversation partner We hypothesised that participants primed with attachments would be more likely to talk about relationship-related issues because this prime would heighten activation of the attachment system and thereby promote awareness of interpersonal factors.

## Materials and methods

### Participants

Participants (N = 98) were recruited using the same first-year psychology student recruitment methods as used for Experiment 1, but were a drawn from a different cohort to Experiment 1. All participants received course credit for participation, and completed written informed consent. Participants were enrolled between February 10, 2019 and April 14, 2019.

### Measures

Measures were the same as for Experiment 1, with the addition of the end-of-life writing task, which was developed for the purposes of this experiment. The writing task instructions were as follows: “*Now*, *still imagining that you’re in the scenario that you just imagined*, *having terminal cancer*. *On the next page I’d like you to imagine that you’re having a conversation with a really close friend or family member*, *someone you could talk about anything with*. *Please write in as much detail as possible what you would say to that person*. *Really try to open up and share your deepest thoughts and feelings around end-of-life*, *and the things you would want to tell that close person in this scenario*.*”*

### Procedure

The procedure began the same as Experiment 1. Participants completed the DASS-21, and then were randomly allocated to either the attachment or control induction. Participants then completed the same end-of-life visualisation. However, instead of being told they may be speaking to a psychologist, participants were instead asked to write what they would want to say in a conversation with someone they are close to, in context of the hypothetical incurable illness scenario. They were encouraged to “really open up” and express their thoughts and feelings around end-of-life. Participants were prompted to continue writing for six minutes. Participants were thanked and debriefed at the end of the session.

The first author was aware of participants’ identities, and these were stored in de-identified forms in the primary datasets prior to being available to other members of the research team.

### Analysis

As with Experiment 1, we estimated a required sample size of at least 35 per cell to achieve an effect size of 0.6 between the two conditions, providing power of 80% to detect a difference between conditions at the 5% significance level. All tests were performed using SPSS Version 25. Descriptive statistics were used to describe the sample, and t-tests and repeated measures ANOVAs were used to test the differences between attachment and control groups on the manipulation checks. To test the relationships between groups, individual differences in attachment security, and willingness to talk about end-of-life, moderation analyses were performed using the PROCESS macro v.3 (Model 2) for SPSS 25. Three moderation analyses were conducted with group (attachment vs control) as the predictor variable, ECR-R attachment and avoidant subscales as the moderator variables, and willingness to talk as the outcome variable. We ran separate moderation analyses with willingness to talk to friends/family, talking to a psychologist, and talking to friends/family/a psychologist as the outcome variable.

The writing task was analysed by identifying the key themes reported in the responses during readings of the texts rather than employing pre-determined constructs, i.e. manifest content analysis [24, 32]. The first author initially read the transcripts and identified themes. A second independent rater (RB) read the scripts and the identified themes were those that were concordant between the two raters. The major themes found were relationship issues, references to self-efficacy, worry for others, optimism and emotional valence. Text was coded for presence (1) or absence (0) of the first four themes, and emotional valence was coded as negative (1) or neutral (0).

## Results and discussion

### Participant characteristics

Participant characteristics are presented in Table 3. Of 98 participants, eight were excluded due to scores over the cut-offs on the DASS-21 Depression and/or Anxiety scores, resulting in 45 participants in each condition. There was a significant difference between the attachment and control groups on ECR-R [*t*(88) = 2.57, *p* = .012], such that the attachment group was higher on the anxious attachment subscale. No other differences were found between groups on participant age or scores on the DASS-21 or the avoidant subscale of the ECR-R. Group means fell within the normal to mild ratings on the DASS-21 Depression, Anxiety and Stress scales.

**Table 3:** Participant characteristics Experiment 2.

	Group	
	Attachment mean (*SD*)	Control mean (*SD*)
Age	20.03 (*4*.*9*)	20.84 (*4*.*9*)
Gender (percentages)	73% female	60% female
DASS-21		
Depression	3.29 (*2*.*2*)	3.20 (*2*.*73*)
Anxiety	4.0 (*2*.*86*)	3.04 (*2*.*47*)
Stress	5.64 (*3*.*16*)	5.18 (*3*.*18*)
ECR-R		
Anxious	3.80 (.*73*)	3.38 (.*82*)
Avoidant	2.89 (*1*.*01*)	2.95 (*1*.*03*)
	Attachment N *(%)*	Control N *(%)*
Education level attained		
Post graduate degree	0	1 *(2*.*2)*
University degree	1 *(2*.*2)*	2 *(4*.*4)*
Technical certificate/diploma, college	2 *(4*.*4)*	4 *(8*.*9)*
Year 12 (the final year of high school in Australia)	42 *(93*.*3)*	38 *(84*.*4)*
Language other than English spoken at home		
No, English only	19 *(42*.*2)*	17 *(37*.*8)*
Yes	26 *(57*.*8)*	28 *(62*.*2)*

*Note—*SD, Standard deviation; DASS-21, Depression Anxiety Stress Scale 21-item; ECR-R (higher scores = higher insecure attachment, score has a minimum of zero and maximum of seven), Experiences in Close Relationships Scale–Revised.

### Manipulation checks

There were no differences between the attachment [*M* = 78.27, *SD* = 17.71] and control [*M* = 74.82, *SD* = 21.79] conditions on vividness of induction [*t*(88) = .823, *p* = .413]. A 2 (Induction Condition; Attachment vs Control) x 2 (Time) ANOVA on closeness ratings revealed a significant main effect for Time [*F*(1,88) = 5.311, *p* = .024, η_p_^2^ = .057], indicating that participants’ reported levels of closeness reduced after the end-of-life visualisation task (see Table 4).

**Table 4:** Experiment 2 means and standard deviations for manipulation checks.

	Group	
	Attachment mean *(SD)*	Control mean (*SD*)
Closeness		
Post-induction	70.42 *(19*.*38)*	69.20 *(20*.*09)*
Post-visualisation	65.58 *(23*.*71)*	63.40 *(26*.*69)*
Distress		
Pre-induction	20.53 *(20*.*53)*	14.91 *(20*.*23)*
Post-induction	16.34 *(16*.*37)*	16.84 *(20*.*87)*
Post-visualisation	43.96 *(24*.*21)*	44.51 *(29*.*97)*
Positivity		
Pre-induction	62.69 *(23*.*95)*	74.58 *(19*.*02)*
Post-induction	73.13 *(20*.*80)*	75.29 *(17*.*65)*
Post-visualisation	46.96 *(22*.*90)*	55.67 *(24*.*11)*

A 2 (Induction Condition) x 3 (Time) ANOVA (with a Greenhouse-Geisser correction to correct for lack of sphericity) on distress ratings indicated a significant main effect for Time [*F*(1.64,143.05) = 77.22, *p* = < .001, η_p_^2^ = .47]. Post-hoc contrasts revealed a significant difference between post-induction and post-visualisation [F(1,87) = 94.38, *p* = < .001, η_p_^2^ = .52], but not between pre-induction and post-induction [F(1,87) = .550, *p* = .460, η_p_^2^ = .006]. This indicates that distress ratings did not differ by group, however both groups increased equally in distress following the end-of-life visualisation.

A 2 (Induction Condition) x 3 (Time) ANOVA (with a Greenhouse-Geisser correction to correct for lack of sphericity) on positivity ratings indicated a significant main effect for Time [*F*(1.787, 157.260) = 55.779, *p* = < .001, η_p_^2^ = .388]. Contrasts revealed significant differences between pre-induction and post-induction [*F*(1,88) = 8.243, *p* = .005, η_p_^2^ = .053], and between post-induction and post-visualisation [*F*(1,88) = 106.522, *p* = < .001, η_p_^2^ = .548]. There was also a significant interaction between positivity and group between pre-induction and post-induction [*F*(1,88) = 6.28, *p* = .014, η_p_^2^ = .067]. This pattern indicated that there was an increase in positivity in the attachment group between pre- and post-induction, relative to controls, however both groups decreased equally in positivity following the end-of-life visualisation.

### Willingness to talk

For the first moderation analysis using talking to friends/family as the outcome variable, the priming condition did not independently predict willingness to talk [*b* = -1.36, *t*(74) = -.605, *p* = .547]. However, the interaction with avoidant attachment style was significant [*F*(1, 74) = 3.09, *p* = .003, change *R*^*2*^ = .026], indicating that more avoidantly attached individuals felt less willing to talk when provided with the attachment prime.

For the second moderation analysis using talking to a psychologist as the outcome variable, the priming condition did not independently predict willingness to talk [*b* = -.75, *t*(74) =.-.299, *p* = .766]. However, the interaction with avoidant attachment style was significant [*F*(1, 74) = 13.35, *p* = .0001, change *R*^*2*^ = .115], indicating again that more avoidantly attached individuals felt less willing to talk when provided with the attachment prime.

**Fig 2:**
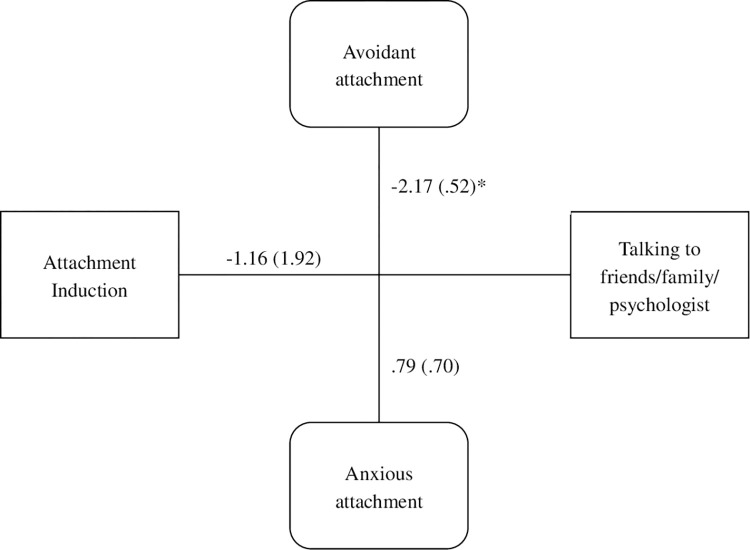
Experiment 2 moderation model of attachment induction, talking about end-of-life, anxious attachment and avoidant attachment.

In the final moderation analysis, see Fig 2, the induction condition did not independently predict willingness to talk [*b* = -1.16, *t*(74) = -.60, *p* = .55]. However, the interaction with avoidant attachment style was significant [*F*(1, 74) = 8.82, *p* < .001, change *R*^*2*^ = .07]. As with Experiment 1, there was no main effect of attachment induction on willingness to talk, but this was dependent on attachment avoidance; those high in attachment avoidance gravitated away from talking to others about end-of-life issues whether or not they had been primed to think about a close attachment figure.

### End-of-life writing task

Five themes were identified from the content analysis: self-efficacy, worry for others, optimism, and relationship issues (see Table 5). A chi-square test indicated that more participants in the attachment condition referenced relationships in their writing task than those in the control condition [*χ*^*2*^(1, *N* = 89) = 5.02, *p* = .025], however the effect size for this relationship was weak [Cramer’s *V* = .238]. For example, a reference to relationship issues included *‘I remember all the good times that we have shared since childhood*, *all the laughter*, *how you have saved me when I did something wrong and how much fun we have had together’*.

**Table 5:** Experiment 2 counts and percentages for presence of themes in participant writing.

	Group				
	Control count	Attachment count	*χ* ^*2*^	*p*	*V*
Self-efficacy presence	22 (49%)	28 (62%)	1.35	.245	.123
Worry for others presence	25 (56%)	26 (58%)	.11	.736	.036
Optimism presence	20 (44%)	23 (51%)	.95	.331	.103
Relationship issues presence	15 (33%)	26 (58%)	5.02	.025*	.238

Note—*Statistically significant. V, Cramer’s V

## General discussion

These experiments examined the role of attachment in simulated end-of-life communication in healthy young people. While across both experiments, the attachment induction did not increase participants’ willingness to talk about end-of-life related concepts, our experiments suggested that avoidantly-attached individuals may be more reluctant to talk about end-of-life issues. Further, participants receiving the attachment induction were more likely to want to talk about their relationship with the person they were talking with. This is consistent with previous literature finding that avoidantly-attached individuals are less likely to engage with others for support and express emotions in difficult situations [33], and are more likely to utilise alternative methods of emotion regulation [34].

We found that avoidantly-attached participants were less likely to be influenced by the attachment induction in being willing to engage in an end-of-life conversation. This accords with the proposition that avoidance attachment tendencies involves hypoactivation of the attachment system when faced with threat, and they tend to distance from others [17]. Consistent with this proposal, numerous studies have found that avoidantly attached people do not respond to attachment priming [22, 24]. Moreover, there are many studies indicating that avoidant attachment tendencies are associated with negative appraisals of relationships [35, 36]. At an applied level, it has been noted that attachment avoidance in end-stage cancer patients is linked to lack of social support [37], and it has been shown that insecure attachment tendencies are associated with poorer psychological adjustment to cancer [38]. Nonetheless, it should be noted that people with avoidant attachment tendencies may also manage this experience by using more independent coping strategies, and in this sense apparent avoidance may not necessarily reflect a maladaptive predisposition [39]. This perspective is supported by evidence that avoidantly attached people are able to engage in self-focused survival strategies in response to threat, which may be adaptive [40]. Self-focused coping strategies and not seeking support from others could be adaptive in the short term for young people given that cancer takes them out of their normal social words, although in the long-term this is unlikely to be beneficial.

Experiment 2 featured the addition of a writing task where participants were asked to write what they would say if they were having a conversation on end-of-life topics with a close friend or family member. Participants in the attachment condition were not observed to be more likely than those in the control group to talk about topics surrounding self-efficacy, worry for others, optimism, or to talk in more or less emotional depth. However, participants receiving the attachment induction were more likely to address topics around relationship issues. This is perhaps due to a priming effect of the attachment induction, placing relationship issues at the front of their minds.

Finally, given the finding that the attachment induction influences the content of participants’ simulated end-of-life conversations, and that attachment avoidance moderates the relationship between the attachment induction and willingness to engage in end-of-life conversations, individual differences in attachment avoidance may also influence the content of end-of-life conversations. This is a question which could be explored in further research, as it could inform the real-world implementation of end-of-life conversations with avoidantly attached patients. For example, conversations on certain topics may require more or less support from healthcare professionals.

There are several limitations to the present studies. The first and most important of these is the nature of the participants used. Participants in the present experiments were generally both physically and psychologically healthy, young, highly educated and mostly female. This limits the generalisability of these findings not only to the general population, but specifically to AYAs who are experiencing their own or others’ end-of-life. However, the use of analogue samples has been used in previous research [41, 42], and is theoretically justified in that attachment has been identified as a variable of interest in previous palliative care research [43–49]. The approach of using analogue samples was taken in the current research program to (a) allow manipulations of factors that would permit causal inferences to be drawn, and (b) permit the necessary pre-clinical studies in an ethically sound way prior to undertaking comparable studies with clinical populations.

## Conclusions

In summary, these findings have potential implications for how patients managing their end-of-life may be assisted. There is often a perception of reluctance for patients to engage in end-of-life conversations, however these discussions can be very important for both practical and psychological reasons. There is increasing recognition that the content of these conversations should be tailored to the needs of the individual [50], and the current findings highlight that one’s attachment tendency may need to be considered in determining the benefit of encouraging end-of-life conversations with others. Petersen and Koehler [46] suggest a careful and gentle approach to discussing palliative topics with avoidantly attached patients; these findings emphasise the potentially important role of psychologists within the multidisciplinary healthcare team in tailoring and supporting end-of-life communication taking these individual attachment tendencies into account [51]. However, it remains unclear whether engaging avoidantly attached individuals in an end-of-life conversation would be felt as aversive or whether they receive the benefits from self-disclosure. These possibilities point to the need for more focused research on the role of attachment theory in understanding end-of-life conversations with AYAs. This will contribute to a scientific understanding of how relational aspects of end-of-life communication and care can be optimised to ensure that the best care can be provided for all young people, regardless of attachment style.
